# Research Progress on the Relationship Between Artificial Sweeteners and Breast Cancer

**DOI:** 10.3390/biomedicines12122871

**Published:** 2024-12-18

**Authors:** Xianqiang Yu, Zeng Yu, Xiaoli Chen, Meijun Liu, Feng Yang, Kenneth C. P. Cheung

**Affiliations:** 1 Qingdao Municipal Hospital, Qingdao 266005, China; yuxianqiang302@126.com; 2 Phenome Research Center, School of Chinese Medicine, Hong Kong Baptist University, Hong Kong, China; zengyu0718@163.com (Z.Y.); xlchenucdavis@gmail.com (X.C.); lmj20210322@163.com (M.L.); 3 The First Affiliated Hospital of Zhejiang University School of Medicine, Hangzhou 310003, China; yf3979988@163.com

**Keywords:** artificial sweeteners, breast cancer, estrogen, aspartame, oxidative stress, gut microbiome

## Abstract

Artificial sweeteners, as low-calorie sugar substitutes, have attracted much attention in recent years, especially in terms of their potential health effects. Although they add almost no calories, studies have shown that artificial sweeteners may affect metabolism by stimulating insulin secretion and changing the intestinal microbiota, increasing the risk of metabolic syndrome and type 2 diabetes. Breast cancer, as the most common cancer in the world, is related to multiple factors such as genetics and hormone levels. The results of studies on artificial sweeteners and breast cancer risk are conflicting, with some showing a positive correlation between the two and others failing to confirm it. Differences in study design, participant characteristics, and the types of sweeteners have led to this ambiguity. Although some studies have focused on mechanisms such as hormone disorders, insulin response, and changes in the intestinal microbiota, further exploration is needed to establish a causal relationship. Our review aims to comprehensively analyze the potential association between artificial sweeteners and breast cancer and its mechanisms, as well as encourage future studies to reveal its long-term health effects.

## 1. Introduction

The development of artificial sweeteners has evolved over the years to meet the demands of consumers seeking low-calorie alternatives to sugar [1,2]. These sweeteners are chemically synthesized to provide sweetness without contributing significant calories to the diet. As scientific knowledge advanced, researchers began developing new methods for chemical synthesis, leading to the creation of more artificial sweeteners with enhanced sweetness profiles and reduced off-tastes. Modern artificial sweeteners are often referred to as “high-intensity sweeteners” because they provide sweetness at much lower concentrations than sugar [3,4,5]. They are used in significantly smaller quantities to achieve the desired sweetness level in food and beverages, contributing negligible calories to the diet.

However, artificial sweeteners have long been a subject of public concern regarding their potential health impacts [6,7,8,9,10]. Artificial sweeteners produce a strong sweet taste by efficiently binding to taste receptor type 1 member 2 (T1R2) and taste receptor type 1 member 3 (T1R3) sweet taste receptors, but most of them cannot be metabolized into energy by the human body. Although they do not directly increase blood sugar levels, studies have shown that they may trigger insulin secretion through taste receptor activation. Long-term intake may lead to insulin resistance and metabolic disorders, increasing the risk of metabolic syndrome and type 2 diabetes [11]. In addition, some artificial sweeteners such as sucralose may change the composition of the intestinal microbiota, reduce beneficial bacteria, promote the growth of harmful bacteria, and then cause metabolic problems such as inflammation, obesity, and insulin resistance [12,13,14]. However, its specific mechanism has not been fully elucidated and further research and verification are needed. Breast cancer is the currently most diagnosed cancer worldwide and the leading cause of death in females. In 2020, 2.2 million breast cancer patients were diagnosed, which led to 685,000 deaths in the same year. Early detection, early diagnosis, and more effective treatments have increased the 5-year survival rate in developed regions to over 80%, while in South Africa, only half of breast cancer patients survive after 5 years [15]. Breast cancer is the most commonly diagnosed cancer globally, with an estimated 2.3 million new cases in 2020, accounting for 11.7% of all cancer diagnoses [16]. It has surpassed lung cancer as the leading cancer worldwide in terms of incidence among females. The highest age-standardized incidence rates (ASIRs) are observed in regions with high socio-demographic indices (SDIs), such as North America and Western Europe, where rates exceed 75 cases per 100,000 individuals. Meanwhile, regions with low SDIs, such as Sub-Saharan Africa, report much lower incidence rates (around 8 cases per 100,000). However, these regions are experiencing relatively faster growth in incidence rates due to increasing urbanization, lifestyle changes, and improved diagnostic capacity. Countries with lower SDIs face unique challenges, including late-stage diagnoses and limited access to advanced treatment options [17]. These factors contribute to a disproportionately higher mortality rate relative to incidence in these regions compared to high-income countries, where survival rates exceed 85% over five years. The primary preventive methods against breast cancer include a healthy diet which limits the consumption of sweet beverages, high-calorie food, alcohol, and processed meat, avoiding being obese, and engaging in more physical exercise [18].

As one might assume, the possible link between artificial sweetener consumption and breast cancer development has garnered particular attention. Numerous epidemiological studies have investigated the potential connection between artificial sweetener intake and breast cancer risk [19,20]. A study by Debras C et al. found that a high intake of aspartame and acesulfame potassium significantly increased the risk of breast cancer. Participants who consumed high doses of artificial sweeteners daily had an incidence of breast cancer that was approximately 13% higher than those who did not consume them [20]. However, the available evidence on the association between artificial sweeteners and breast cancer remains inconclusive and often contradictory. Some studies have reported an increased risk of breast cancer with higher artificial sweetener consumption, while others have found no significant association [19,21].

Genetic factors (such as BRCA1 and BRCA2 gene mutations) and hormone levels (especially estrogen) are the main risk factors for breast cancer [22,23]. Although artificial sweeteners are not directly associated with genetic mutations, they may indirectly interfere with estrogen balance and increase breast cancer risk by affecting metabolic function and insulin sensitivity, especially when consumed over a long period of time. Specifically, it promotes the occurrence of cancer by changing insulin sensitivity, fat metabolism, and intestinal microbiota, hence inducing inflammation, insulin resistance, obesity, and other problems.

Further research is essential to elucidate the mechanistic pathways and establish a clear association between artificial sweetener consumption and breast cancer risk. This review aims to critically examine the current state of evidence on the relationship between artificial sweeteners and breast cancer, exploring the underlying mechanisms proposed to explain this association.

## 2. Potential Mechanisms Underlying the Association

### 2.1. Hormonal Disruption

**Figure 1:**
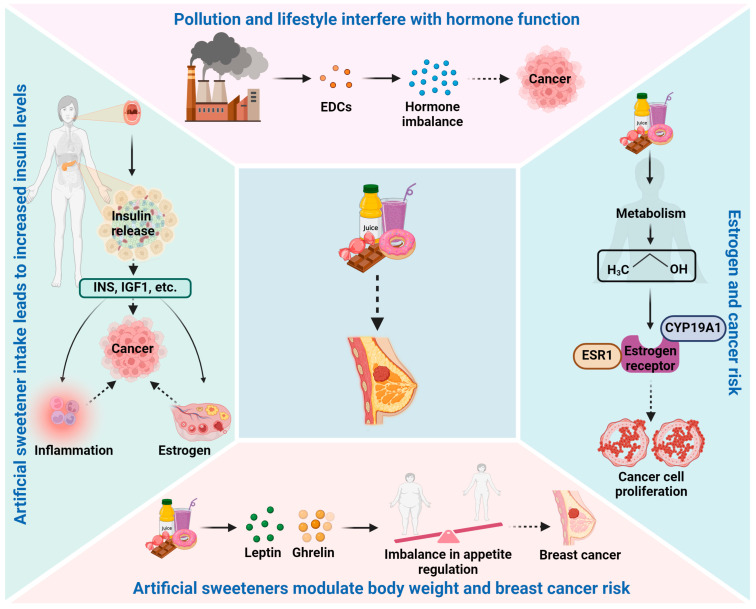
Artificial sweeteners affect hormonal pathways in breast cancer. Methanol and formaldehyde produced by artificial sweeteners in the body may interfere with the estrogen signaling pathway, leading to hormone imbalance and breast cancer cell proliferation. They may also further increase breast cancer risk by affecting insulin and IGF levels and the balance of leptin and ghrelin.

One of the primary concerns with artificial sweeteners is their potential impact on hormone regulation. Certain artificial sweeteners, such as aspartame, undergo metabolic processes that produce methanol, which can be converted to formaldehyde [24,25,26,27]. This process has raised concerns about potential estrogen receptor interactions and hormonal imbalances that could promote breast cancer development. Nevertheless, the actual significance of these metabolic byproducts in human health is a topic of ongoing research and debate. The potential link between artificial sweeteners and hormonal disturbances in breast cancer has been a subject of growing interest and research. Hormonal disturbances, particularly involving estrogen and insulin pathways, play a crucial role in breast cancer development and progression [28,29,30]. Artificial sweeteners, which are widely used as sugar substitutes due to their low-calorie content, have raised concerns regarding their impact on hormone regulation. This elaborate discussion explores the potential mechanisms through which artificial sweeteners may influence hormonal disturbances in breast cancer (Figure 1).

Estrogen is a key hormone in breast cancer development, particularly in hormone receptor-positive breast cancers. Some artificial sweeteners, such as aspartame, contain phenylalanine, which can be metabolized into methanol and further converted into formaldehyde. Formaldehyde is known to be a xenoestrogen, an estrogen-like compound that can mimic the actions of natural estrogen in the body. Xenoestrogens have the potential to disrupt the delicate balance of estrogen signaling, leading to hormonal imbalances that may promote breast cancer cell proliferation [29]. Additionally, studies have shown that aspartame consumption can influence estrogen receptor expression and activity in breast cancer cells, suggesting a potential direct effect on estrogen-related pathways [31]. These findings raise questions about the potential role of aspartame in modulating estrogen signaling and contributing to hormonal disturbances in breast cancer.

Insulin and insulin-like growth factors (IGFs) are essential in regulating glucose metabolism and cell growth [32,33]. Some studies have suggested that artificial sweeteners, particularly those that taste sweet, can trigger insulin release in anticipation of glucose intake, even though they lack calories [34]. The repeated consumption of sweeteners without a corresponding rise in glucose levels may lead to dysregulated insulin signaling, potentially impacting insulin sensitivity and increasing insulin and IGF levels in the blood. Elevated insulin and IGF levels have been associated with increased breast cancer risk, as these hormones can promote cell proliferation and inhibit apoptosis (programmed cell death) [35]. Furthermore, insulin and IGFs may influence breast cancer progression by interacting with other growth factors and receptors involved in cell growth and survival. Therefore, the potential impact of artificial sweeteners on insulin and IGF pathways is an important area of investigation regarding their role in hormonal disturbances and breast cancer development.

Leptin and ghrelin are hormones involved in appetite regulation and energy balance. Leptin is produced by adipose tissue and helps regulate body weight by signaling satiety and reducing food intake [36,37]. Ghrelin, on the other hand, is produced mainly in the stomach and stimulates appetite, promoting food intake [38,39]. Some studies have suggested that artificial sweeteners may disrupt the balance of leptin and ghrelin, leading to altered hunger and satiety signals [40]. Dysregulation of these hormones could influence eating behaviors, food preferences, and overall caloric intake, potentially impacting body weight. Obesity is a well-established risk factor for postmenopausal breast cancer, and any artificial sweetener-induced changes in leptin and ghrelin levels may have implications for breast cancer risk through obesity-related mechanisms.

Artificial sweeteners may interact with other environmental factors, such as endocrine-disrupting chemicals (EDCs), to influence hormonal disturbances [41]. EDCs are chemicals that can interfere with hormone function and signaling, potentially affecting breast cancer risk [42,43]. Some studies have suggested that artificial sweeteners may act synergistically with certain EDCs to enhance hormonal disruptions. For example, in vitro studies have shown that aspartame can potentiate the estrogenic effects of bisphenol A (BPA), a well-known EDC [44]. This suggests that simultaneous exposure to artificial sweeteners and EDCs may have additive or synergistic effects on hormonal disturbances, potentially impacting breast cancer risk.

The potential mechanisms linking artificial sweeteners to hormonal disturbances in breast cancer are multifaceted and complex. While some studies have reported associations between artificial sweetener consumption and hormonal changes, the evidence is still limited and inconclusive. The effects of artificial sweeteners may vary depending on the specific type and dose of sweetener, individual metabolism, and overall dietary patterns. It is essential to recognize that much of the existing evidence comes from preclinical studies and observational data, which cannot establish causality definitively. The complexity of the human body’s response to artificial sweeteners necessitates further rigorous research, including well-designed human intervention trials, to establish a clearer understanding of their impact on hormonal disturbances and breast cancer risk.

### 2.2. Microbiome Alterations

**Figure 2:**
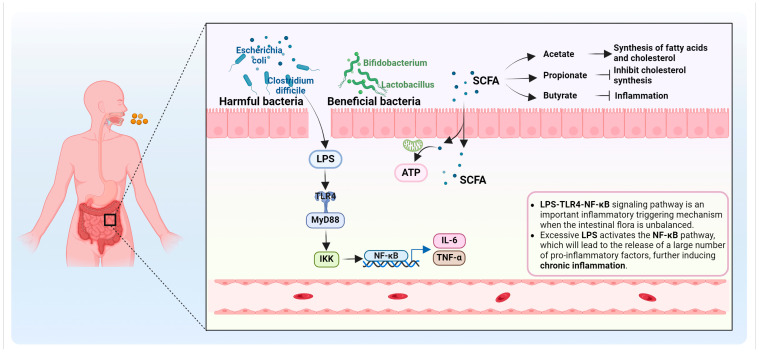
Mechanisms by which artificial sweeteners affect breast cancer risk via the gut microbiome. Artificial sweeteners affect the composition of the intestinal microbiome, resulting in a decrease in beneficial bacteria and an increase in harmful bacteria, thereby reducing the production of SCFAs. The reduction in SCFAs triggers intestinal inflammation, impaired intestinal barrier function, and increased intestinal permeability, causing endotoxins (such as LPSs) to enter the blood and trigger a systemic inflammatory response. The LPS-TLR4-NF-κB signaling pathway represents an inflammatory cascade activated by LPSs released from pathogenic gut bacteria. Upon binding to TLR4, LPSs triggers the MyD88-dependent pathway, leading to the activation of NF-κB signaling, which subsequently induces the expression of pro-inflammatory cytokines such as IL-6 and TNF-α. This chronic inflammatory response is closely associated with the initiation and progression of breast cancer.

Emerging evidence indicates that artificial sweeteners may influence the gut microbiome, potentially impacting various physiological processes [45,46,47]. Dysbiosis of the gut microbiota has been implicated in various health conditions, including cancer [48,49,50]. The potential link between artificial sweeteners and microbiome alterations in breast cancer is an emerging area of research with significant implications for human health. The gut microbiome refers to the diverse community of microorganisms residing in the gastrointestinal tract, including bacteria, viruses, and fungi. These microorganisms play a crucial role in various physiological processes, such as nutrient metabolism, immune system regulation, and protection against pathogens. Disruptions in the gut microbiome have been associated with several health conditions, including metabolic disorders, inflammatory diseases, and potentially breast cancer (Figure 2).

Research suggests that artificial sweeteners can influence the composition and diversity of the gut microbiome [51,52,53]. Several studies have reported changes in the abundance of certain gut bacterial species following artificial sweetener consumption. For example, aspartame, saccharin, and sucralose have been shown to alter the relative abundance of specific bacterial taxa in the gut, including decreasing the proportion of beneficial bacteria and increasing the abundance of potentially harmful ones. One of the crucial functions of the gut microbiome is to ferment dietary fibers and complex carbohydrates that are not digestible by human enzymes. During this fermentation process, beneficial gut bacteria produce short-chain fatty acids (SCFAs), such as acetate, propionate, and butyrate [54,55]. SCFAs play a significant role in maintaining gut health, regulating inflammation, and influencing various physiological processes. Artificial sweeteners, particularly non-caloric sweeteners, do not provide fermentable substrates for gut bacteria [53]. Consequently, the lack of available dietary fibers may reduce the production of SCFAs by the gut microbiome, potentially impacting gut health and immune function. Reduced SCFA production has been associated with increased gut inflammation and compromised gut barrier function, both of which have been implicated in breast cancer risk. In addition, dysbiosis and reduced SCFA production may contribute to increased gut permeability, often referred to as “leaky gut”. A compromised gut barrier can allow for the translocation of bacterial endotoxins, such as lipopolysaccharides (LPSs), from the gut lumen into the bloodstream [56]. This condition, known as endotoxemia, triggers a systemic inflammatory response. Chronic inflammation and elevated levels of circulating endotoxins have been associated with various health conditions, including breast cancer. Inflammatory processes and endotoxin-related pathways have been implicated in breast cancer development and progression.

The gut microbiota is involved in the metabolism of estrogens, influencing their effects on breast tissue. Microbial species, such as Bacteroides, Firmicutes, and Lactobacillus, can impact estrogen levels by producing enzymes like beta-glucuronidase, which deconjugates estrogen metabolites, potentially increasing estrogenic activity in the body. Lactobacillus has been associated with reduced estrogenic activity and may lower breast cancer risk by metabolizing estrogen into inactive forms [57]. Bacteroides and Firmicutes, however, might increase the bioavailability of active estrogen, enhancing the growth-promoting effects of estrogens in breast tissue [58]. This relationship highlights the role of microbial diversity in determining estrogenic activity, a key factor in breast cancer risk. The immune system is a crucial player in cancer progression, and gut microbiota can modulate immune responses that either inhibit or promote breast cancer. The gut microbiome influences immune cell populations, including T cells, macrophages, and dendritic cells, which are involved in the recognition and destruction of cancer cells. Dysbiosis (an imbalance in the microbiota composition) can lead to an altered immune response that promotes tumor progression. Certain species, like Faecalibacterium prausnitzii, have anti-inflammatory effects and may help prevent cancer by maintaining immune homeostasis [59]. Conversely, Enterococcus and Bacteroides can promote a pro-inflammatory environment that facilitates cancer development through the activation of inflammatory cytokines and immune suppression [60]. Inflammation can drive carcinogenesis through mechanisms such as increased cell proliferation and DNA damage, leading to tumor growth. Thus, microbial-induced immune modulation can create a tumorsupportive or tumorsuppressive environment depending on the microbial composition. The gut microbiome also influences immune checkpoints, such as PD-1 and CTLA-4, which can regulate the immune system’s ability to detect and destroy tumor cells. Microbial species have been shown to modulate these checkpoints, altering the immune response to cancer cells. Bifidobacterium and Lactobacillus species can stimulate immune responses that make tumors more vulnerable to immune system attacks by modulating checkpoint pathways and enhancing anti-tumor immunity [61]. On the other hand, some bacteria may activate immune checkpoints that inhibit the immune response against tumors, allowing cancer cells to evade immune surveillance [62]. This suggests that microbial types can influence whether cancer cells escape immune destruction, further emphasizing the microbiome’s role in breast cancer progression.

Emerging research highlights the pivotal role of the gut and breast microbiomes in influencing cancer risk. Specific bacterial strains can modulate cellular signaling pathways, potentially promoting or suppressing cell proliferation. Key studies have identified microbiome dysbiosis as a contributing factor to carcinogenesis. The study by Romanos-Nanclares et al. highlights how aspartame, a common artificial sweetener, may contribute to breast cancer risk through microbial changes [63]. Although specific bacterial strains were not directly identified in this study, it provides evidence that alterations in gut microbiota composition can influence pathways linked to inflammation and estrogen metabolism—key factors in breast cancer development. This finding underscores the need to investigate specific bacteria modulated by artificial sweeteners to establish a clear link with oncogenesis. Ranjbar et al. also explored the role of Fusobacterium nucleatum, a bacterium linked to colorectal cancer, as a potential trigger for tumorigenesis [64]. This bacterium promotes cell proliferation by interacting with the host’s immune signaling pathways and epithelial cells, creating a pro-inflammatory environment. Although its direct role in breast cancer is yet to be validated, its mechanisms provide a foundation for hypothesizing similar microbial influences in breast tissue. Alizadehmohajer et al. elaborated how dysbiosis impacts women’s cancers, including breast cancer [65]. The research identifies microbial imbalances that modulate estrogen metabolism, oxidative stress, and inflammatory pathways. For instance, bacteria capable of converting estrogen precursors into active forms may exacerbate hormone-driven cancers, highlighting their role in cell proliferation and tumor development. These additions will provide a comprehensive discussion of the microbiome’s role, tying artificial sweeteners to breast cancer risk through microbial intermediaries.

The potential mechanisms underlying the link between artificial sweeteners and microbiome alterations in breast cancer are complex and multifaceted. While several studies have reported associations between artificial sweetener consumption and gut microbiome changes, the evidence is still preliminary and requires further investigation. Until more conclusive evidence is available, it is advisable to consume artificial sweeteners in moderation and prioritize a diet rich in fiber and whole foods to support a healthy gut microbiome. A balanced diet, regular physical activity, and overall healthy lifestyle habits remain crucial factors in breast cancer prevention and overall well-being.

### 2.3. Psychological and Behavior Factors

It is essential to consider psychological and behavioral aspects when examining the relationship between artificial sweeteners and breast cancer. Some studies have suggested that individuals consuming artificial sweeteners may overcompensate by increasing overall caloric intake or developing unhealthy eating habits, which could influence cancer risk indirectly [23]. The potential link between artificial sweeteners and psychological and behavioral factors in breast cancer is an area of growing interest and research. Psychological and behavioral factors can significantly impact overall health, including breast cancer risk and prognosis. Artificial sweeteners have been scrutinized for their potential effects on mental well-being and eating behaviors [66].

Psychological factors, such as stress, anxiety, and depression, have been associated with breast cancer risk and outcomes. Chronic psychological stress can lead to dysregulation of the hypothalamic–pituitary–adrenal [67] axis, resulting in elevated cortisol levels [68]. Increased cortisol, a stress hormone, has been linked to inflammation and immune system suppression, both of which can affect cancer development and progression. Artificial sweeteners have been reported to modulate the release of neurotransmitters, including serotonin and dopamine, in the brain [69,70]. Serotonin is a neurotransmitter involved in mood regulation, and low serotonin levels have been associated with an increased risk of depression. Some studies have suggested that artificial sweeteners may reduce serotonin levels in the brain, potentially impacting mental well-being. Moreover, the intense sweetness of artificial sweeteners can activate reward pathways in the brain, triggering the release of dopamine, a neurotransmitter associated with pleasure and reward. Repeated activation of the reward system by artificial sweeteners may influence food preferences and eating behaviors, potentially leading to addictive-like behavior toward sweet-tasting foods. These psychological aspects may contribute to stress and emotional eating patterns, which could impact body weight and breast cancer risk. Psychological and behavioral factors play a significant role in breast cancer risk and outcomes, and addressing these factors is essential for overall health and well-being. Adopting a balanced and varied diet, managing stress, engaging in regular physical activity, and seeking support for mental health are all crucial components of a healthy lifestyle that may reduce breast cancer risk.

### 2.4. Oxidative Stress and Inflammatory Response

Some artificial sweeteners have been found to interfere with the normal function of mitochondria, causing damage to their electron transport chain and resulting in excessive production of reactive oxygen species (ROS). When ROS levels are too high, the cell’s antioxidant system cannot effectively remove these free radicals, which in turn causes oxidative stress and exacerbates mitochondrial dysfunction [71]. This accumulation of oxidative stress promotes the survival and proliferation of breast cancer cells, as ROS can activate multiple signaling pathways associated with cancer progression [72]. In addition, artificial sweeteners may further enhance oxidative stress by inhibiting the activity of key antioxidant enzymes, such as glutathione peroxidase and superoxide dismutase. This oxidative environment provides the basis for tumor cells to undergo biological changes that favor proliferation and invasion through ROS-induced signaling pathways.

At the same time, artificial sweeteners promote the release of inflammatory factors and increase ROS generation by activating inflammatory signaling pathways such as NF-kappaB (NF-κB), further promoting oxidative stress. Excessive ROS can cause oxidative damage to DNA, including base oxidation and double-strand breaks, increasing the risk of gene mutation [73]. These DNA lesions interfere with normal replication and repair processes. If not repaired in time, they may lead to cell cycle arrest or apoptosis. However, some cells may survive through apoptosis escape or mutation mechanisms, forming an unstable genome, thereby promoting the occurrence and development of tumors.

Artificial sweeteners may also directly interact with intestinal microorganisms, change the composition of intestinal flora, and induce a systemic chronic low-grade inflammatory response [74]. Intestinal microbial imbalance can lead to impaired intestinal barrier function, increase the transport of endogenous substances, induce the body’s immune response, and then aggregate inflammatory cells, release pro-inflammatory factors such as tumor necrosis factor α (TNF-α) and interleukin (IL-6), and increase the risk of tumor occurrence [75]. Chronic low-grade inflammation not only promotes cell proliferation and inhibits cell apoptosis but also changes the cell microenvironment, thereby further promoting the occurrence and development of breast cancer. Continuous inflammatory signals activate cell proliferation-related signaling pathways (such as PI3K/Akt and MAPK), leading to the proliferation and survival of cancer cells in breast tissue while triggering immunosuppression in the tumor microenvironment, promoting the growth and metastasis of cancer cells [76].

### 2.5. Artificial Sweeteners and Metabolic Changes

Minimal differences were found between natural non-nutritive sweeteners and artificial sweeteners regarding postprandial glucose and insulin levels [77]. Notably, sucralose does not stimulate the release of insulin, GLP-1, or GIP [78]. Non-nutritive artificial sweeteners are associated with higher fat oxidation and lower carbohydrate oxidation compared to water controls and regular sugar-sweetened beverages, suggesting potential health benefits [79].

Interestingly, there are conflicting data on whether artificial sweeteners can be effectively used to prevent obesity and type 2 diabetes. Generally, randomized controlled trials support the use of artificial sweeteners for managing obesity and type 2 diabetes, while prospective cohort studies do not. It is hypothesized that participants in the cohort studies may be more likely to be obese or diabetic and tend to consume more without careful monitoring. This behavior may stem from the misconception that foods containing artificial sweeteners have fewer calories than their actual values, contributing to the observed inconsistencies [80].

Further research is needed to clarify the exact role of artificial sweeteners. To date, there is not enough experimental evidence indicating that artificial sweeteners cause more harmful metabolic changes compared to their natural sugar counterparts, at least in the short term.

Nevertheless, in young adults, ten weeks of sucralose consumption has shown to alter gut microbiota, increasing the area under the curve of glucose levels and serum insulin levels, though neither were statistically significant [12]. Both low doses (0.0003 mg/mL) and high doses (0.3 mg/mL) of sucralose reduce probiotic gut microbiome abundance (*Lachnoclostridium* and *Lachnospiraceae*) in mice and increase potential pathogens, including *Tenacibaculum*, *Ruegeria*, and *Staphylococcus*, as well as diabetes-associated *Allobaculum*, while a middle dosage group (0.003, 0.03 mg/mL) showed milder changes [13]. Sucralose can also induce chronic inflammation in mice gut and liver [14]. Considering that estrogen, one of the most critical breast-cancer-associated hormones produced from the liver and pre-existing gut dysbiosis, is strongly associated with tissue inflammation and tumor cell dissemination [81], it is credible to believe that artificial sweeteners like sucralose can affect breast cancer indirectly by altering gut microbiota; however, uncertainty remains about how and to what extent the negative effects of sucralose in the gut microbiome can translate to breast cancer treatments and patient survival. More long-term cohort studies are needed to investigate the relationship among the artificial sweeteners, gut microbiome, inflammation, liver, estrogen, and breast cancer.

### 2.6. Artificial Sweeteners Inhibit T Cell-Mediated Immune Response

The oral administration of aspartame in mice can induce pro-inflammatory cytokines such as TNF-α, IL-6, and IL-1β. Notably, high doses of aspartame further stimulate TNF-α and IL-1β levels compared to lower doses [82]. TNF-α is linked to tumor progression, metastasis, angiogenesis, and tumor resistance to immunotherapies. Similarly, elevated levels of plasma IL-1β and tumor-derived IL-1β are associated with increased tumor invasiveness and poor prognosis in breast cancer [83].

Functional impairments in CD8+ T effector cells have been observed in sucralose-treated mice. Weaker T cell-mediated tumor rejection and a significant delay in tumor response both indicate the potential inhibitory effects of sucralose on T cell activity. Sucralose supplementation can inhibit pro-inflammatory IFN-γ-producing CD4+ T cells and Tregs, thereby mitigating T cell-mediated autoimmune responses [84]. In breast cancer, higher expressions of CD4+ and CD8+ T cells are associated with better patient survival, while elevated levels of Tregs contribute to poorer prognosis [85]. A study by Margolis et al. examined the effects of artificial sweeteners on immune markers in humans [86]. The study found that a high consumption of artificial sweeteners altered inflammatory markers, which could indirectly affect T cell-mediated immunity. These effects were more pronounced in individuals with existing immune dysfunction or autoimmune conditions. Human microbiome studies have shown that artificial sweeteners like aspartame can alter gut microbiota composition, which is closely linked to immune modulation [52]. A study published by Suez et al. found that saccharin intake in humans led to significant changes in gut microbiota, influencing immune responses. These changes may inhibit T cell activation, affecting the body’s ability to respond to pathogens and potentially promoting an inflammatory environment conducive to diseases like cancer. These studies suggest that artificial sweeteners, by altering the gut microbiota and immune cell function, may impair T cell activity and overall immune response, which could have implications for autoimmune diseases, cancer progression, and immunotherapy outcomes.

The complex role of sucralose in regulating T cell responses requires further extensive studies to clarify its potential negative effects in breast cancer patients, as well as its implications for treatments targeting other autoimmune diseases.

## 3. Strengths and Limitations

This review comprehensively examines the literature on the association between artificial sweeteners and breast cancer risk, synthesizing the results of epidemiological studies and emphasizing potential mechanisms such as hormonal disruption and microbiome alterations. The discussion of various sweeteners and their metabolic pathways provides a multi-perspective view and emphasizes the need for further research, especially in terms of causality and long-term health effects.

Despite the strengths of the review, the evidence remains uncertain and often contradictory, and many studies are observational, limiting the establishment of causality. Individual response differences, the heterogeneity of study designs, and potential confounding factors make it difficult to draw clear conclusions. In addition, many studies rely on animal models or in vitro experiments, which may not truly reflect human physiological responses. Publication bias and differences in participant characteristics may also affect the overall understanding. Therefore, future randomized controlled trials and dose–response studies are essential, especially for an in-depth exploration of the effects of different sweetener types and their mixtures. Most research on the relationship between artificial sweeteners and breast cancer risk has indeed been conducted in specific populations or under controlled settings, and these studies can have significant limitations when generalizing to broader populations. Given these limitations, while the findings of studies on artificial sweeteners and breast cancer risk provide valuable insights, the results must be interpreted cautiously. Further research with more diverse populations, controlled settings, and long-term follow-up is needed to draw more definitive conclusions about the relationship between artificial sweeteners and breast cancer risk. Selection bias occurs when the participants chosen for the study are not representative of the general population. A study that does not include a diverse population might lead to an over- or underestimation of the relationship between artificial sweeteners and breast cancer. In addition, many studies do not distinguish between varying doses of artificial sweeteners. High-dose exposures may have different effects than low-dose ones, and without understanding the specific quantities consumed, it is difficult to draw firm conclusions about the relationship between artificial sweeteners and cancer risk. Studies that do not account for dose–response relationships might not accurately assess the risks. Acknowledging these limitations will strengthen the overall interpretation and help in making evidence-based recommendations regarding artificial sweetener consumption.

## 4. Future Directions

Future research should aim to clarify the molecular pathways linking artificial sweeteners to breast cancer. This includes investigating potential disruptions in signaling pathways like estrogen receptor modulation, oxidative stress, and inflammation. Advanced technologies such as RNA sequencing and proteomics could help uncover these pathways. It is essential to examine whether genetic predispositions to breast cancer, such as mutations in BRCA1/2 and other high-penetrance genes, modulate the effects of artificial sweeteners. Additionally, exploring how artificial sweeteners impact gene expression through epigenetic mechanisms such as DNA methylation and histone modification could provide critical insights. The gut microbiome is emerging as a mediator between artificial sweetener intake and systemic health outcomes. Studies should focus on whether gut microbiota changes induced by sweeteners promote breast cancer by influencing estrogen metabolism or chronic inflammation. Large-scale longitudinal studies are crucial to understanding the association between artificial sweeteners and breast cancer risk. Such studies should account for variables like sweetener type, duration of exposure, dosage, and demographic differences, including age, ethnicity, and family history. Breast cancer encompasses diverse subtypes with varying prognoses and treatment responses. Investigating whether artificial sweeteners exert distinct effects on hormone receptor-positive, HER2-positive, or triple-negative breast cancers could guide personalized dietary recommendations. By addressing these directions, the scientific community can build a comprehensive understanding of how artificial sweeteners influence breast cancer risk, ultimately improving prevention and management strategies.

The research on artificial sweeteners and breast cancer risk has generated considerable interest, but translating these findings into actionable recommendations for healthcare professionals, policymakers, and the public requires careful consideration of the evidence and its limitations. Translating research findings into recommendations involves balancing the existing evidence with the potential for future discoveries. Healthcare professionals, policymakers, and the public need to remain informed while taking a cautious, evidence-based approach to artificial sweeteners and cancer risk. It is essential to emphasize healthy lifestyle choices, including proper nutrition and physical activity, as the most impactful ways to reduce cancer risk.

## 5. Conclusions

The relationship between artificial sweeteners and breast cancer remains unclear, and existing research results are contradictory. Some studies have pointed out that artificial sweeteners may increase the risk of breast cancer through mechanisms such as hormone disorders, changes in insulin response, and changes in intestinal microbiota, but these hypotheses still need further clinical verification. Although regulators believe that most people’s intake is within a safe range, in the absence of more conclusive evidence, a moderate use of artificial sweeteners combined with a balanced diet, regular exercise, and preventive screening is still a safe choice to maintain health. As the use of artificial sweeteners continues to rise, understanding their long-term health effects is crucial to public health policies and consumer decisions, and future research should further clarify their specific association with breast cancer.
